# Joint Interference Alignment and Power Allocation for *K*-User Multicell MIMO Channel through Staggered Antenna Switching

**DOI:** 10.3390/s18020380

**Published:** 2018-01-28

**Authors:** Poongundran Selvaprabhu, Sunil Chinnadurai, Md.Abdul Latif Sarker, Moon Ho Lee

**Affiliations:** Department of Electronics and Information Engineering, Chonbuk National University, Jeonju 561-756, Korea; poongundran@jbnu.ac.kr (P.S.); sunilkcsss@jbnu.ac.kr (S.C); latifsarker@jbnu.ac.kr (M.L.S.)

**Keywords:** blind interference alignment, multiple-input multiple-output (MIMO), Gaussian interference channel, reconfigurable multimode antenna

## Abstract

In this paper, we characterise the joint interference alignment (IA) and power allocation strategies for a *K*-user multicell multiple-input multiple-output (MIMO) Gaussian interference channel. We consider a MIMO interference channel with blind-IA through staggered antenna switching on the receiver. We explore the power allocation and feasibility condition for cooperative cell-edge (CE) mobile users (MUs) by assuming that the channel state information is unknown. The new insight behind the transmission strategy of the proposed scheme is premeditated (randomly generated transmission strategy) and partial cooperative CE MUs, where the transmitter is equipped with a conventional antenna, the receiver is equipped with a reconfigurable multimode antenna (staggered antenna switching pattern), and the receiver switches between preset *T* modes. Our proposed scheme assists and aligns the desired signals and interference signals to cancel the common interference signals because the received signal must have a corresponding independent signal subspace. The capacity for a *K*-user multicell MIMO Gaussian interference channel with reconfigurable multimode antennas is completely characterised. Furthermore, we show that the proposed *K*-user multicell MIMO scheduling and *K*-user *L*-cell CEUs partial cooperation algorithms elaborate the generalisation of *K*-user IA and power allocation strategies. The numerical results demonstrate that the proposed intercell interference scheme with partial-cooperative CE MUs achieves better capacity and signal-to-interference plus noise ratio (SINR) performance compared to noncooperative CE MUs and without intercell interference schemes.

## 1. Introduction

Interference alignment (IA) is one of the most prominent techniques in wireless communication systems because of their increase in size of wireless network, complexity and challenges encountered [1]. The achievable rate and channel capacity region for a two-cell Z multiple-input-multiple-output (MIMO) interference channel and *K*-user relay aided multiple-input-single-output (MISO) interference channel are characterized with blind-IA and imperfect channel knowledge schemes, respectively [2,3]. Topological interference management scheme for MIMO relay channel and two-cell two-hop MIMO half-duplex relay channel schemes require limited channel information to align the interfering signals. IA is the finest approach for time-varying MIMO interference networks, as it requires only limited resources such as time, frequency and number of users [4,5]. Some special cases require global channel knowledge, such as perfect and imperfect channel information. The retrospective-IA, blind-IA and linear precoding techniques require channel temporal correlation structure or private information from the transmitter side. To align the interference signals in an efficient manner, private information retrieval retrieves the *K*-user channel values at the receiver side by using databases to recognize the desired message index values [6,7,8,9]. In particular, ergodic IA scheme required past (delayed) channel information (feedback) from the transmitter for effective IA strategies [10]. Authors in [11,12] reported that a dark-blind IA scheme consider staggered antenna switching approach (transmitter equipped with M antennas and K receivers) for vector broadcast channel to solve the interference problems. It was also shown that dark-blind IA through staggered antenna switching method provide better-IA scheme for MISO and single-input-single-output (SISO). interference channels. In [13,14], they discussed the joint optimization for multiple-relay MIMO channel and channel auto correlation for MISO broadcast channels, where the achievable degree of freedom (DoF) is determined based on the total number of independent interference-free signal dimensions for multiple-relay systems. The authors in [15] focus on the energy-efficiency of a load-adaptive Massive MIMO system, by considering user location and distribution on dynamic power allocation strategy. The blind-IA approach established on staggered reconfigurable antenna modes to generate short-term channel fluctuation patterns that are exploited by the transmitter is briefly deliberated in [16]. The effect of robustness antenna selection for spatial multiplexing and Alamouti space-time interference alignment (STIA) techniques for asynchronous cooperative systems are briefly discussed, respectively in [17,18]. In [19], the antenna switching pattern (reconfigurable antennas) scheme is extensively used to align the desired and interference signals automatically in a downlink broadcast channel. Relay-assisted interference network and the achievable DoF for MIMO Gaussian broadcast channels assists with aligning the interference signals in an efficient manner under non-cooperative transmission strategy [20,21].

In this paper, we propose new IA and power allocation schemes for a Gaussian interference channel where each receiver is equipped with reconfigurable antennas (staggered antenna switching pattern) capable of switching between *T* preset modes. The authors in [2] consider two cell MIMO interference channels by focusing two main scenarios (*L* = 2, *K* = 2) such as (1) mobile user (UE1) located very far (cell edge user) from the base station (BS1) and (2) the mobile user (UE2) located very close to the base station (cell center user). In the existing scheme from [20], where the matrix dimension $\tilde{P}=[\tilde{p_{1}},\tilde{p_{2}},\ldots ,\tilde{P_{K}}]$ is designed based on $\tilde{P}=\left[1_{K\times K}-I_{K\times K},A_{\left((1/2)K-1\times (K-1)\times K\right)}\right]$ and the matrix premeditated (randomly generated transmission strategy), but the beamforming vector $\left\{u_{1}^{k},u_{2}^{k}\right\}$ is evaluated through multiplying the switching vector of individual channel matrix, for example $\left\{u_{1}^{k},u_{2}^{k}\right\}=[\tilde{p_{1}}*\tilde{p_{2}}]$. Due to the novelty of the proposed scheme, the matrix dimension $\tilde{P}=[\tilde{p_{1}},\tilde{p_{2}},\ldots ,\tilde{P_{K}}]$ is constructed through $\tilde{P}=B^{(1/2)((K+1)+(K-1))\times K}$, and both switching matrix and beamforming vector are premeditated (randomly generated transmission strategy). Moreover, we extend our approach to joint IA and power allocation strategies, for (*L* = 3 cells, *K* = 5 users) is characterised in Section 3 and Section 5. The alignment scheme is based on mild assumptions on the channel coherence structure, and we consider a low configurable switching antenna to switch between two preset (*T* = 2) modes. Since the proposed scheme does not require cooperation at the transmitter, the key idea behind the proposed scheme is to align the interference signals by creating a short-term channel fluctuation pattern at the transmitter side. This new insight behind the proposed scheme is that any vector of a user aligned at one unintended receiver (by cancelling similar dimensional subspaces) cannot be aligned at the other unintended receiver. In addition, we consider a multicell MIMO partial-cooperative network and power allocation strategies to improve cell-edge user performance. We completely characterise the *K*-user, multicell MIMO Gaussian interference channel through the staggered antenna switching pattern in the absence of channel state information (CSI). The base stations (BSs) use the channel fluctuation pattern information to adapt their environment and communicate strategies.

### 1.1. Summary of Contributions

The main contributions of this paper are summarized as follows.

- The novelty of this research is that the interference signal subspace is aligned by considering a randomly generated transmission strategy.
- We propose a partially cooperative cell-edge (CE) mobile users (MUs) scheme and two-tier transmitter beamforming strategy at BSs, to minimize the interuser interference (IUI) leakage (*K* = 5 users) and intercell interference (ICI) leakage (*L* = 3 cells) among the cells.
- Since our proposed algorithm considers a low-cost reconfigurable antenna switch among only (T=2) preset modes, we generalise the IA and power allocation approaches for the *K*-user MIMO Gaussian interference channel by using the staggered antenna switching pattern in the absence of CSI.
- Most importantly, the idea behind the proposed scheme is to assist and align the desired and interference signals by cancelling out the similar dimensional subspace signals because any vector aligned at one undesired receiver cannot be aligned at the other unintended receiver.
- The proposed scheme splits the users into three groups cell-center user (CCU), cell-median user (CMU), and cell-edge user (CEU). We also extended our approach to the partial cooperation between CE MUs and with ICI schemes for a multicell MIMO network; the data sharing between cooperative CE MUs and ICI schemes drastically improves the overall system performance.
- In addition, our result applies to a downlink *K*-user multicell MIMO Gaussian interference channel with reconfigurable antennas at the receiver and considers the effect of the partial cooperation of CE MUs on interference channel scenarios with no CSI knowledge.
- Numerically, we show that the *K*-user multicell MIMO scheduling and *K*-user *L*-cell CE MUs cooperation algorithms improve the overall system performance for the *K*-user interference channel.
- The capacity for the proposed cooperative CE MUs multimode staggered antenna switching and with ICI schemes achieve better system performance compared to the noncooperative CE MUs and without ICI schemes.

### 1.2. Organisation

The rest of this paper is organised as follows. Section 2 introduces the system model and definitions for the K=5 users and L=1 cell MIMO Gaussian interference channels. In Section 3, the proposed scheme for staggered multimode antenna switching is discussed. Section 4 presents the multicell MIMO cooperative network that assists in improving CEU performance. The generalisation of the proposed scheme that facilitates partial cooperation between CE MUs and power analysis for cooperative CE MUs is briefly discussed in Section 5. Section 6 describes the *K*-user, *L*-multicell MIMO Gaussian interference channel through staggered antenna switching, (CEUs) Partial Cooperation and power analysis. Section 7 presents the numerical results, and we conclude the paper in Section 8.

## 2. System Model and Definitions

**Figure 1:**
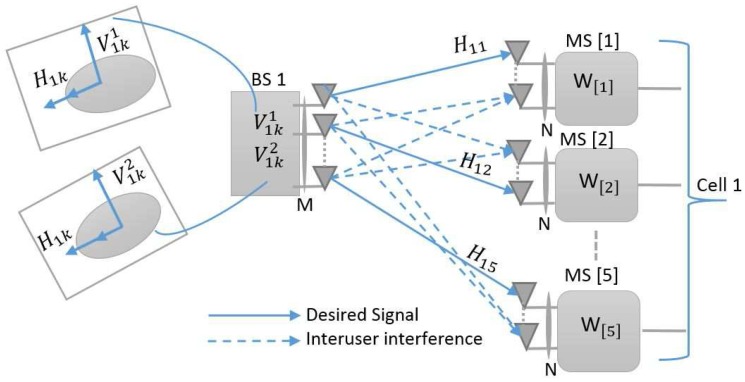
Multiple-input-multiple-output Gaussian interference channel for five users per cell (K=5 and L=1).

The MIMO Gaussian interference channel model for five users per cell (K=5 and L=1) is depicted in Figure 1. For any user *K*, the transmission strategy is predetermined based on the reconfigurable multimode antenna and the receiver switch between *T* preset modes. Here, we consider a low-cost reconfigurable multimode antenna switch between only two modes T=2.

**Lemma** **1.** For reconfigurable multimode antenna switching pattern matrix dimensions, $\tilde{P_{K}}$ always exists and is described as follows: $\tilde{P_{K}}=[\tilde{p_{1}},\tilde{p_{2}},\ldots ,\tilde{P_{K}}]=B^{(1/2)((K+1)+(K-1))\times K}$, such as the matrix $P_{K}$ column vectors must have the same dimension as $\tilde{P_{K}}$ has a full column rank.

Considering a fully connected K=5 MUs MIMO Gaussian interference channel, since each independent user transmits two independent (desired) symbols $s_{1}^{\left[K\right]}$ and $s_{2}^{\left[K\right]}$ over eight (interference) time slots, one of the possible reconfigurable multimode antenna patterns is given below.

**Proof.**  We can easily prove that $\tilde{P_{K}}$ is a matrix that satisfies the above conditions; we know that the transmission strategy of the proposed scheme is predetermined. The staggered antenna switching pattern for K=5 users, the matrix $\tilde{P_{K}}$ with one possible choice, is defined as follows:

(1) $$\begin{array}{c} P^{T}=\left[\begin{array}{ccccc} 1 & 1 & 2 & 2 & 2\\ 2 & 1 & 2 & 2 & 1\\ 1 & 2 & 2 & 2 & 1\\ 2 & 2 & 2 & 1 & 1\\ 2 & 1 & 2 & 1 & 1 \end{array}\right]. \end{array}$$

The proposed scheme uses a low-cost reconfigurable antenna, which operates only in the two switching modes proposed in [1,2]. Here, we will design the antenna switching pattern for receiver *k*. At the end of the second time slot, we switch the antenna from mode 1 to mode 2 for receiver 1:

(2) $$\begin{array}{c} P_{1}^{T}=\left[\begin{array}{ccccc} 1 & 1 & 2 & 2 & 2 \end{array}\right]. \end{array}$$

The channel matrix designed based on transmitter *k* and receiver 1 can be expressed as

(3) $$\begin{array}{c} H_{1k}=diag\left(\left[h_{1k}\left(1\right)h_{1k}\left(1\right)h_{1k}\left(2\right)h_{1k}\left(2\right)h_{1k}\left(2\right)\right]\right). \end{array}$$

At the end of the third time slot, we switch the antenna from mode 1 to mode 2 for receiver 2:

(4) $$\begin{array}{c} P_{2}^{T}=\left[\begin{array}{ccccc} 2 & 1 & 2 & 2 & 1 \end{array}\right]. \end{array}$$

The channel matrix designed based on transmitter *k* and receiver 2 can be expressed as

(5) $$\begin{array}{c} H_{2k}=diag\left(\left[h_{2k}\left(2\right)h_{2k}\left(1\right)h_{2k}\left(2\right)h_{2k}\left(2\right)h_{2k}\left(1\right)\right]\right). \end{array}$$

For receiver 3, we switch the antenna mode twice. At the end of the first time slot, we switch the antenna from mode 1 to mode 2 and at the end of the fourth time slot, the antenna is switched back to mode 1:

(6) $$\begin{array}{c} P_{3}^{T}=\left[\begin{array}{ccccc} 1 & 2 & 2 & 2 & 1 \end{array}\right]. \end{array}$$

The channel matrix designed based on transmitter *k* and receiver 3 can be expressed as

(7) $$\begin{array}{c} H_{3k}=diag\left(\left[h_{3k}\left(1\right)h_{3k}\left(2\right)h_{3k}\left(2\right)h_{3k}\left(2\right)h_{3k}\left(1\right)\right]\right). \end{array}$$

At the end of the third time slot, we switch the antenna from mode 2 to mode 1 for receiver 4:

(8) $$\begin{array}{c} P_{4}^{T}=\left[\begin{array}{ccccc} 2 & 2 & 2 & 1 & 1 \end{array}\right]. \end{array}$$

The channel matrix designed based on transmitter *k* and receiver 4 can be expressed as

(9) $$\begin{array}{c} H_{4k}=diag\left(\left[h_{4k}\left(2\right)h_{4k}\left(2\right)h_{4k}\left(2\right)h_{4k}\left(1\right)h_{4k}\left(1\right)\right]\right). \end{array}$$

For receiver 5, we switch the antenna mode twice. At the end of the first time slot, we switch the antenna from mode 2 to mode 1 and the end of the fourth time slot, the antenna is switched back to mode 2:

(10) $$\begin{array}{c} P_{5}^{T}=\left[\begin{array}{ccccc} 2 & 1 & 2 & 1 & 1 \end{array}\right]. \end{array}$$

The channel matrix designed based on transmitter *k* and receiver 5 can be expressed as

(11) $$\begin{array}{c} H_{5k}=diag\left(\left[h_{5k}\left(2\right)h_{5k}\left(1\right)h_{5k}\left(2\right)h_{5k}\left(1\right)h_{5k}\left(1\right)\right]\right). \end{array}$$

For *K* = 5 users, each user transmits two symbols $s_{1}^{\left[k\right]}\;\mathrm{and}\;s_{2}^{\left[k\right]}$; hence, the beamforming vectors are given by

(12) $$\begin{array}{c} s_{2}^{1}=s_{1}^{2}=\left[\begin{array}{ccccc} 0 & 1 & 1 & 1 & 0 \end{array}\right]\\ s_{2}^{2}=s_{1}^{3}=\left[\begin{array}{ccccc} 0 & 0 & 1 & 1 & 1 \end{array}\right]\\ s_{2}^{3}=s_{1}^{4}=\left[\begin{array}{ccccc} 1 & 1 & 1 & 0 & 0 \end{array}\right]\\ s_{2}^{4}=s_{1}^{5}=\left[\begin{array}{ccccc} 1 & 0 & 0 & 1 & 1 \end{array}\right]\\ s_{2}^{5}=s_{1}^{1}=\left[\begin{array}{ccccc} 1 & 1 & 0 & 0 & 1 \end{array}\right]. \end{array}$$

 ☐

## 3. Multimode Antenna Switching

**Figure 2:**
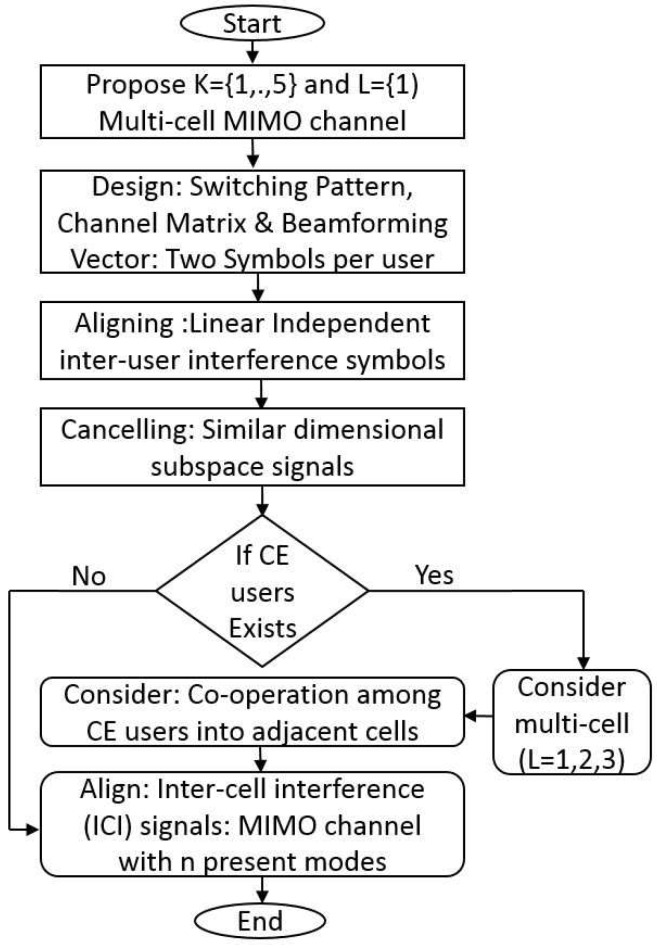
Step-by-step procedure of the proposed optimisation framework.

In this section, we elaborate single cell *K* = 5 user MIMO interference channel with two preset modes (*T* = 2 antenna switching modes) and we also explained IA cases for *K* = 1, 3, 5 user cases. In our proposed scheme, we consider special cases, such as *K* = 5 user IA scheme in Section 3 and *L* = 3 cells CEU cooperation scheme in Section 5. These special cases are essential for the manuscript to understand the general case and algorithms in Section 6. The essential steps in the proposed optimisation framework scheme are shown in Figure 2. We need to observe if receiver 1 can decode its two desired symbols and eight interference symbols by multiplying corresponding beamforming vectors and channel vectors, respectively, and this is briefly discussed as follows.

It is also essential to observe that the receiver can decode in total ten symbols (desired and interference symbols) over the first time slot and we discuss this as follows. We examine each received signal and decode the desired and interference signals; since $y_{1}$ has two desired signals and eight interference signals, the antenna switching pattern for receiver 1 is given in Table 1 and the received signal vector for receiver 1 is expressed as follows,

(13) $$\begin{array}{cc} y_{1}\;\;\;\; & =\underset{\mathrm{Desired}\;\mathrm{signal}}{\underset{︸}{H_{11}x_{1}}}+\underset{\mathrm{Interference}\;+\;\mathrm{Noise}}{\underset{︸}{\;\sum_{k=2}^{5}H_{1k}x_{k}+Z_{1}}}=\left[\begin{array}{cc} h_{11}\left(1\right) & 0\\ h_{11}\left(1\right) & h_{11}\left(1\right)\\ 0 & h_{11}\left(2\right)\\ 0 & h_{11}\left(2\right)\\ h_{11}\left(2\right) & 0 \end{array}\right]\;\;\left[\begin{array}{c} s_{1}^{\left[1\right]}\\ s_{2}^{\left[1\right]} \end{array}\right]+Z_{1}\\ & +\;\;\left[\begin{array}{cccccccc} 0 & 0 & 0 & h_{13}\left(1\right) & h_{14}\left(2\right) & h_{14}\left(2\right) & h_{15}\left(2\right) & h_{15}\left(2\right)\\ h_{12}\left(1\right) & 0 & 0 & h_{13}\left(2\right) & h_{14}\left(2\right) & 0 & 0 & h_{15}\left(1\right)\\ h_{12}\left(2\right) & h_{12}\left(2\right) & h_{13}\left(2\right) & h_{13}\left(2\right) & h_{14}\left(2\right) & 0 & 0 & 0\\ h_{12}\left(2\right) & h_{12}\left(2\right) & h_{13}\left(2\right) & 0 & 0 & h_{14}\left(1\right) & h_{15}\left(1\right) & 0\\ 0 & h_{12}\left(1\right) & h_{13}\left(1\right) & 0 & 0 & h_{14}\left(1\right) & h_{15}\left(1\right) & h_{15}\left(1\right) \end{array}\right]\left[\begin{array}{c} s_{1}^{\left[2\right]}\\ s_{2}^{\left[2\right]}\\ s_{1}^{\left[3\right]}\\ s_{2}^{\left[3\right]}\\ s_{1}^{\left[4\right]}\\ s_{2}^{\left[4\right]}\\ s_{1}^{\left[5\right]}\\ s_{2}^{\left[5\right]} \end{array}\right]. \end{array}$$

The channel matrix and the transmit signal vector denoted by H, $x_{k}$ respectively. To produce the two-dimensional desired signal subspace, we must align the eight-dimensional subspace and cancel the interference subspace signals of similar dimensions. The matrix $R_{1}$ has two desired symbols and interference symbols, and the interference symbols that align in the same dimensional subspace are cancelled:

(14) $$\begin{array}{c} R_{1}=\left[\begin{array}{cccccccccc} h_{11}\left(1\right) & 0 & 0 & 0 & 0 & h_{13}\left(1\right) & h_{14}\left(2\right) & h_{14}\left(2\right) & h_{15}\left(2\right) & h_{15}\left(2\right)\\ h_{11}\left(1\right) & h_{11}\left(1\right) & h_{12}\left(1\right) & 0 & 0 & h_{13}\left(2\right) & h_{14}\left(2\right) & 0 & 0 & h_{15}\left(1\right)\\ 0 & h_{11}\left(2\right) & h_{12}\left(2\right) & h_{12}\left(2\right) & h_{13}\left(2\right) & h_{13}\left(2\right) & h_{14}\left(2\right) & 0 & 0 & 0\\ 0 & h_{11}\left(2\right) & h_{12}\left(2\right) & h_{12}\left(2\right) & h_{13}\left(2\right) & 0 & 0 & h_{14}\left(1\right) & h_{15}\left(1\right) & 0\\ h_{11}\left(2\right) & 0 & 0 & h_{12}\left(1\right) & h_{13}\left(1\right) & 0 & 0 & h_{14}\left(1\right) & h_{15}\left(1\right) & h_{15}\left(1\right) \end{array}\right]. \end{array}$$

From above equation R, interfering symbols $(h_{11}$ and $h_{12})$, $(h_{12}$ and $h_{13})$, $(h_{14}$ and $h_{15})$ are naturally aligned in the same direction. At receiver 1, the transmitter signals are aligned in the interference signal subspace; the interference symbols aligned in the same dimensional subspace are cancelled and the aligned sets are constructed as follows:

(15) $$\begin{array}{c} \left[\begin{array}{c} s_{2}^{\left[1\right]}=s_{1}^{\left[2\right]};\Rightarrow \;H_{11}s_{2}^{\left[1\right]}V_{11}\;\in \;\mathrm{span}\left(H_{12}s_{1}^{\left[2\right]}V_{12}\right)\\ s_{2}^{\left[2\right]}=s_{1}^{\left[3\right]};\Rightarrow \;H_{12}s_{2}^{\left[2\right]}V_{12}\;\in \;\mathrm{span}\left(H_{13}s_{1}^{\left[3\right]}V_{13}\right)\\ s_{2}^{\left[4\right]}=s_{1}^{\left[5\right]};\Rightarrow \;H_{14}s_{2}^{\left[4\right]}V_{14}\;\in \;\mathrm{span}\left(H_{15}s_{1}^{\left[5\right]}V_{15}\right) \end{array}\right]. \end{array}$$

After cancelling the interference symbols that align in the same dimensional subspace, full-rank matrix R is written as follows:

(16) $$\begin{array}{c} R_{1}=\left[\begin{array}{ccccccc} h_{11}\left(1\right) & 0 & 0 & h_{13}\left(1\right) & h_{14}\left(2\right) & h_{14}\left(2\right) & h_{15}\left(2\right)\\ h_{11}\left(1\right) & h_{11}\left(1\right) & 0 & h_{13}\left(2\right) & h_{14}\left(2\right) & 0 & h_{15}\left(1\right)\\ 0 & h_{11}\left(2\right) & h_{12}\left(2\right) & h_{13}\left(2\right) & h_{14}\left(2\right) & 0 & 0\\ 0 & h_{11}\left(2\right) & h_{12}\left(2\right) & 0 & 0 & h_{14}\left(1\right) & 0\\ h_{11}\left(2\right) & 0 & h_{12}\left(1\right) & 0 & 0 & h_{14}\left(1\right) & h_{15}\left(1\right) \end{array}\right]. \end{array}$$

What remains to be shown is that the 5×7 full-rank matrix R contains the two-dimensional desired signal subspaces and five-dimensional interference signal subspaces. By cancelling the three-dimensional interference signals aligned in the same dimensional subspace, receiver 1 can achieve a 2/7 normalised DoF.

We need to observe that receiver 3 can decode its two desired symbols and eight interference symbols by multiplying corresponding beamforming vectors and channel vectors, respectively, as briefly discussed below. The antenna switching pattern for receiver 3 is given in Table 2 and the received signal vector for receiver 3 is expressed as follows,

(17) $$\begin{array}{cc} y_{3}\;\;\;\; & =\underset{\mathrm{Desired}\;\mathrm{signal}}{\underset{︸}{H_{33}x_{3}\;}}+\underset{\mathrm{Interference}\;+\;\mathrm{Noise}}{\underset{︸}{\sum_{k=1}^{2}\sum_{k=4}^{5}H_{3k}x_{k}+Z_{3}}}\;\;=\;\;\left[\begin{array}{cc} 0 & h_{33}\left(1\right)\\ 0 & h_{33}\left(2\right)\\ h_{33}\left(2\right) & h_{33}\left(2\right)\\ h_{33}\left(2\right) & 0\\ h_{33}\left(1\right) & 0 \end{array}\right]\;\;\left[\begin{array}{c} s_{1}^{\left[3\right]}\\ s_{2}^{\left[3\right]} \end{array}\right]+Z_{3}\\ & +\;\;\left[\begin{array}{cccccccc} h_{31}\left(1\right) & 0 & 0 & 0 & h_{34}\left(2\right) & h_{34}\left(2\right) & h_{35}\left(2\right) & h_{35}\left(2\right)\\ h_{31}\left(1\right) & h_{31}\left(1\right) & h_{32}\left(1\right) & 0 & h_{34}\left(2\right) & 0 & 0 & h_{35}\left(1\right)\\ 0 & h_{31}\left(2\right) & h_{32}\left(2\right) & h_{32}\left(2\right) & h_{34}\left(2\right) & 0 & 0 & 0\\ 0 & h_{31}\left(2\right) & h_{32}\left(2\right) & h_{32}\left(2\right) & 0 & h_{34}\left(1\right) & h_{35}\left(1\right) & 0\\ h_{31}\left(2\right) & 0 & 0 & h_{32}\left(1\right) & 0 & h_{34}\left(1\right) & h_{35}\left(1\right) & h_{35}\left(1\right) \end{array}\right]\left[\begin{array}{c} s_{1}^{\left[1\right]}\\ s_{2}^{\left[1\right]}\\ s_{1}^{\left[2\right]}\\ s_{2}^{\left[2\right]}\\ s_{1}^{\left[4\right]}\\ s_{2}^{\left[4\right]}\\ s_{1}^{\left[5\right]}\\ s_{2}^{\left[5\right]} \end{array}\right]. \end{array}$$

The matrix $R_{3}$ has two desired symbols and interference symbols:

(18) $$\begin{array}{c} R_{3}=\left[\begin{array}{cccccccccc} 0 & h_{33}\left(1\right) & h_{31}\left(1\right) & 0 & 0 & 0 & h_{34}\left(2\right) & h_{34}\left(2\right) & h_{35}\left(2\right) & h_{35}\left(2\right)\\ 0 & h_{33}\left(2\right) & h_{31}\left(1\right) & h_{31}\left(1\right) & h_{32}\left(1\right) & 0 & h_{34}\left(2\right) & 0 & 0 & h_{35}\left(1\right)\\ h_{33}\left(2\right) & h_{33}\left(2\right) & 0 & h_{31}\left(2\right) & h_{32}\left(2\right) & h_{32}\left(2\right) & h_{34}\left(2\right) & 0 & 0 & 0\\ h_{33}\left(2\right) & 0 & 0 & h_{31}\left(2\right) & h_{32}\left(2\right) & h_{32}\left(2\right) & 0 & h_{34}\left(1\right) & h_{35}\left(1\right) & 0\\ h_{33}\left(1\right) & 0 & h_{31}\left(2\right) & 0 & 0 & h_{32}\left(1\right) & 0 & h_{34}\left(1\right) & h_{35}\left(1\right) & h_{35}\left(1\right) \end{array}\right]. \end{array}$$

From the above equation for $R_{3}$, interfering symbols $(h_{33}$ and $h_{32})$, $(h_{31}$ and $h_{32})$, $(h_{34}$ and $h_{35})$ are naturally aligned in the same direction. At receiver 3, the transmitter signals are aligned in the interference signal subspace; the same dimensional subspace is cancelled and the aligned sets are constructed as follows:

(19) $$\begin{array}{c} \left[\begin{array}{c} s_{1}^{\left[3\right]}=s_{2}^{\left[2\right]};\Rightarrow \;H_{33}s_{1}^{\left[3\right]}V_{33}\;\in \;\mathrm{span}\left(H_{32}s_{2}^{\left[2\right]V_{32}}\right)\\ s_{2}^{\left[1\right]}=s_{1}^{\left[2\right]};\Rightarrow \;H_{31}s_{2}^{\left[1\right]}V_{31}\;\in \;\mathrm{span}\left(H_{32}s_{1}^{\left[2\right]V_{32}}\right)\\ s_{2}^{\left[4\right]}=s_{1}^{\left[5\right]};\Rightarrow \;H_{34}s_{2}^{\left[4\right]}V_{34}\;\in \;\mathrm{span}\left(H_{35}s_{1}^{\left[5\right]V_{35}}\right) \end{array}\right]. \end{array}$$

From the above matrix, the signals that align in the same dimensional subspace are cancelled. After cancelling the interference symbols that align in the same dimensional subspace, the 5×7 full-rank matrix $R_{3}$ is written as follows:

(20) $$\begin{array}{c} R_{3}=\left[\begin{array}{ccccccc} 0 & h_{33}\left(1\right) & h_{31}\left(1\right) & 0 & h_{34}\left(2\right) & h_{34}\left(2\right) & h_{35}\left(2\right)\\ 0 & h_{33}\left(2\right) & h_{31}\left(1\right) & h_{31}\left(1\right) & h_{34}\left(2\right) & 0 & h_{35}\left(1\right)\\ h_{33}\left(2\right) & h_{33}\left(2\right) & 0 & h_{31}\left(2\right) & h_{34}\left(2\right) & 0 & 0\\ h_{33}\left(2\right) & 0 & 0 & h_{31}\left(2\right) & 0 & h_{34}\left(1\right) & 0\\ h_{33}\left(1\right) & 0 & h_{31}\left(2\right) & 0 & 0 & h_{34}\left(1\right) & h_{35}\left(1\right) \end{array}\right]. \end{array}$$

Finally, we need to observe if receiver 5 can decode its two desired symbols and eight interference symbols by multiplying corresponding beamforming vectors and channel vectors. respectively, briefly discussed as follows.

We examine each received signal and decode the desired and interference signals. $y_{5}$ has two desired signals and eight interference signals; the antenna switching pattern for receiver 5 is given in Table 3 and the received signal vector for receiver 5 is expressed as follows,

(21) $$\begin{array}{cc} y_{5}\;\;\;\; & =\underset{\mathrm{Desired}\;\mathrm{signal}}{\underset{︸}{H_{55}x_{5}}}+\underset{\mathrm{Interference}\;+\;\mathrm{Noise}}{\underset{︸}{\sum_{k=1}^{4}H_{5k}x_{k}+Z_{5}}}\;\;=\;\;\left[\begin{array}{cc} h_{55}\left(2\right) & h_{55}\left(2\right)\\ 0 & h_{55}\left(1\right)\\ 0 & 0\\ h_{55}\left(1\right) & 0\\ h_{55}\left(1\right) & h_{55}\left(1\right) \end{array}\right]\;\;\left[\begin{array}{c} s_{1}^{\left[5\right]}\\ s_{2}^{\left[5\right]} \end{array}\right]+Z_{5}\\ & +\;\;\left[\begin{array}{cccccccc} h_{51}\left(1\right) & 0 & 0 & 0 & 0 & h_{53}\left(1\right) & h_{54}\left(2\right) & h_{54}\left(2\right)\\ h_{51}\left(1\right) & h_{51}\left(1\right) & h_{52}\left(1\right) & 0 & 0 & h_{53}\left(2\right) & h_{54}\left(2\right) & 0\\ 0 & h_{51}\left(2\right) & h_{52}\left(2\right) & h_{52}\left(2\right) & h_{53}\left(2\right) & h_{53}\left(2\right) & h_{54}\left(2\right) & 0\\ 0 & h_{51}\left(2\right) & h_{52}\left(2\right) & h_{52}\left(2\right) & h_{53}\left(2\right) & 0 & 0 & h_{54}\left(1\right)\\ h_{51}\left(2\right) & 0 & 0 & h_{52}\left(1\right) & h_{53}\left(1\right) & 0 & 0 & h_{54}\left(1\right) \end{array}\right]\left[\begin{array}{c} s_{1}^{\left[1\right]}\\ s_{2}^{\left[1\right]}\\ s_{1}^{\left[2\right]}\\ s_{2}^{\left[2\right]}\\ s_{1}^{\left[3\right]}\\ s_{2}^{\left[3\right]}\\ s_{1}^{\left[4\right]}\\ s_{2}^{\left[4\right]} \end{array}\right]. \end{array}$$

From the above equation for $R_{5}$, interfering symbols $(h_{55}$ and $h_{54})$, $(h_{51}$ and $h_{52})$, $(h_{52}$ and $h_{53})$ are naturally aligned in the same direction. At receiver 5, the transmitter signals are aligned in the interference signal subspace; the interference symbols aligned in the same dimensional subspace are cancelled out and the aligned sets are constructed as follows:

(22) $$\begin{array}{c} \left[\begin{array}{c} s_{1}^{\left[5\right]}=s_{2}^{\left[4\right]};\;\Rightarrow \;H_{55}s_{1}^{\left[5\right]}V_{55}\;\in \;\mathrm{span}\left(H_{54}s_{2}^{\left[4\right]V_{54}}\right)\\ s_{2}^{\left[1\right]}=s_{1}^{\left[2\right]};\;\Rightarrow \;H_{51}s_{2}^{\left[1\right]}V_{51}\;\in \;\mathrm{span}\left(H_{52}s_{1}^{\left[2\right]V_{52}}\right)\\ s_{2}^{\left[2\right]}=s_{1}^{\left[3\right]};\;\Rightarrow \;H_{52}s_{2}^{\left[2\right]}V_{52}\;\in \;\mathrm{span}\left(H_{53}s_{1}^{\left[3\right]V_{53}}\right) \end{array}\right], \end{array}$$

(23) $$\begin{array}{c} R_{5}=\left[\begin{array}{cccccccccc} h_{55}\left(2\right) & h_{55}\left(2\right) & h_{51}\left(1\right) & 0 & 0 & 0 & 0 & h_{53}\left(1\right) & h_{54}\left(2\right) & h_{54}\left(2\right)\\ 0 & h_{55}\left(1\right) & h_{51}\left(1\right) & h_{51}\left(1\right) & h_{52}\left(1\right) & 0 & 0 & h_{53}\left(2\right) & h_{54}\left(2\right) & 0\\ 0 & 0 & 0 & h_{51}\left(2\right) & h_{52}\left(2\right) & h_{52}\left(2\right) & h_{53}\left(2\right) & h_{53}\left(2\right) & h_{54}\left(2\right) & 0\\ h_{55}\left(1\right) & 0 & 0 & h_{51}\left(2\right) & h_{52}\left(2\right) & h_{52}\left(2\right) & h_{53}\left(2\right) & 0 & 0 & h_{54}\left(1\right)\\ h_{55}\left(1\right) & h_{55}\left(1\right) & h_{51}\left(2\right) & 0 & 0 & h_{52}\left(1\right) & h_{53}\left(1\right) & 0 & 0 & h_{54}\left(1\right) \end{array}\right]. \end{array}$$

From the above matrix, we observe that the signals that align in the same dimensional subspace are cancelled. After cancelling the interference symbols that align in the same dimensional subspace, the 5×7 full-rank matrix $R_{3}$ is obtained as follows:

(24) $$\begin{array}{c} R_{5}=\left[\begin{array}{ccccccc} h_{55}\left(2\right) & h_{55}\left(2\right) & h_{51}\left(1\right) & 0 & 0 & h_{53}\left(1\right) & h_{54}\left(2\right)\\ 0 & h_{55}\left(1\right) & h_{51}\left(1\right) & h_{51}\left(1\right) & 0 & h_{53}\left(2\right) & h_{54}\left(2\right)\\ 0 & 0 & 0 & h_{51}\left(2\right) & h_{52}\left(2\right) & h_{53}\left(2\right) & h_{54}\left(2\right)\\ h_{55}\left(1\right) & 0 & 0 & h_{51}\left(2\right) & h_{52}\left(2\right) & 0 & 0\\ h_{55}\left(1\right) & h_{55}\left(1\right) & h_{51}\left(2\right) & 0 & h_{52}\left(1\right) & 0 & 0 \end{array}\right]. \end{array}$$

**Figure 3:**
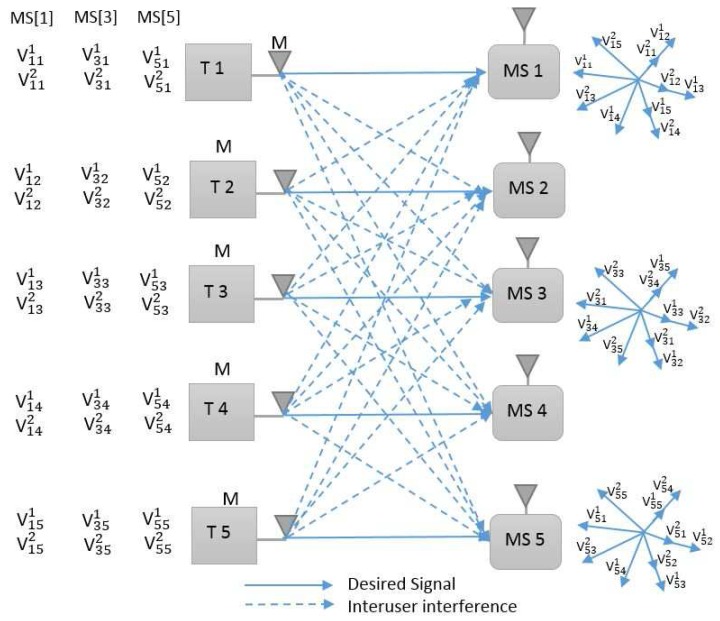
Description for the single-cell five-user multiple-input-multiple-output Gaussian interference channel with two preset modes, i.e., mobile station [1, 3, 5] interference alignment.

The IA for *K* = 1,3,5 users and *L* = 1 cell is illustrated in Figure 3.

## 4. Multicell MIMO Cooperative Network

**Figure 4:**
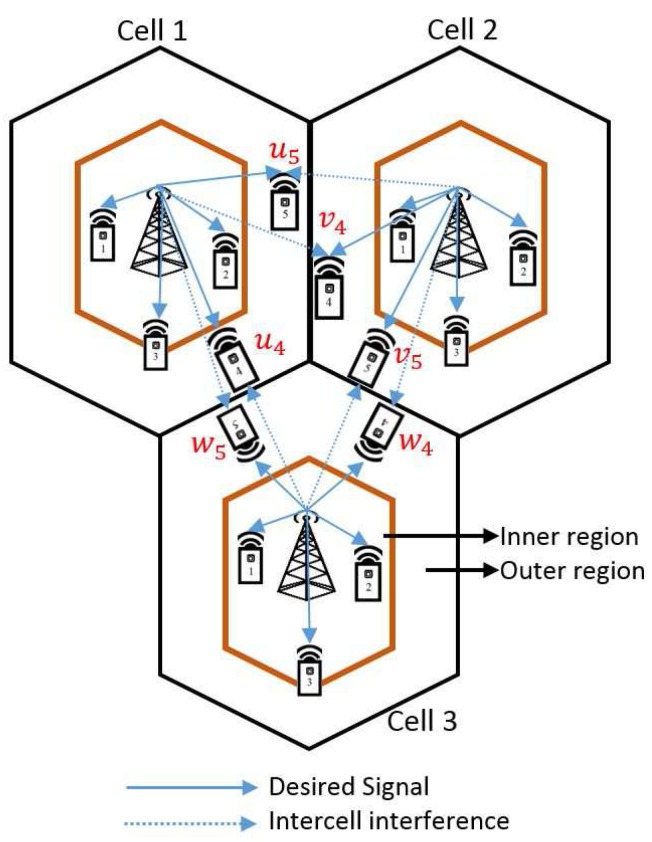
Multiple-input-multiple-output Gaussian interference channel for five users per cell (*K* = 5 and *L* = 3), MUs: {1, 2} cell-centre users, three cell-median user and {4, 5} cell-edge users.

In this section, we focus on the multicell MIMO network by considering the partial cooperation among CE MUs. Since we focus on CE cooperation, we consider the frequency reuse technique to use the same frequency after a certain distance in the wireless system. BS cooperation involves the distribution of control, transmission of data signals, CSI, and precoders through wired backhaul links to synchronise transmitters and MUs. The BSs use this information to adapt their environment and communication strategies to the desired channel conditions. We investigate the multiuser partial cooperation in a multicell environment scenario to improve CEU performance. The signal-to interference-plus-noise ratio (SINR) determines the transmission rate of each MU. We consider partial-cooperative transmission among CEUs for three BSs (L=3) to improve the SINR by jointly transmitting one user at a time, which is illustrated in Figure 4. This type of BS cooperation methodology is reasonable because the BSs are connected through a high-speed wired backhaul that exchanges information among them to maximise the overall system performance. Such a fully cooperative downlink MIMO Gaussian interference channel network leads to the maximum sum rate and throughput; the overall cost is increased due to the large amount of global CSI information exchanges between MUs and BSs.

### 4.1. Non-Cooperation between Users

Under normal operation, we consider the noncooperative transmission scheme between MUs, such as {CCUs, CMUs and CEUs}. The $\left({\mathrm{SINR}}_{\mathrm{nc}}\right)$ for the downlink MIMO Gaussian interference channel network MUs is expressed as follows:

(25) $$\begin{array}{c} C_{\mathrm{nc}}=\log_{2}\left(1+\lambda \;{\mathrm{SINR}}_{\mathrm{nc}}\right). \end{array}$$

The capacity for MUs in bits/s/Hz under the noncooperative transmission scheme is expressed using the Shannon capacity formula, where λ is the determined **SINR** gap between the theoretical and practical coding schemes.

### 4.2. Cooperation between Cell-Edge Users (CEUs)

In general, full cooperation between the users leads to a high sum rate and throughput; the user located near the CE suffers co-channel interference from neighbour cells. Full cooperation assists to increase cost efficiency owing to large amount of information such as (CSI, transmitter, and precoding information) exchange between the users and BSs. However, full cooperation between the CCUs, CMUs, and CEUs users typically generates high complexity and imposes a large load on backhaul links. We consider only the cooperation between CEUs to improve cost efficiency, exchanging large information and high complexity at BSs. We consider the CEUs’ cooperation to the neighbouring CEUs because ${\mathrm{SINR}}_{\mathrm{coop}}$ for the downlink MIMO Gaussian interference channel network will be dependent on the proposed CEUs’ cooperation scheme, The capacity of MUs under the CEUs cooperation in bits/s/Hz is expressed as

(26) $$\begin{array}{c} C_{\mathrm{coop}}=\alpha \log_{2}\left(1+\lambda \;{\mathrm{SINR}}_{\mathrm{coop}}\right), \end{array}$$

where α defines the proportion of resource sharing allocation among the CEU that cooperates. We consider $\alpha =\frac{1}{2}$ as the resource fairness of our proposed scheme. The cooperation among users in CEs and the neighbouring CEs is considered for an **SINR** improvement, by sharing the available resources among the adjacent CE users.

The throughput of individual CEUs is considered as $\alpha =\frac{1}{2}$ of the actual capacity of the downlink MIMO Gaussian interference channel cooperation scheme, as shown in Equation (43), considering λ=1 in Equations (29) and (43) for the low-SINR regime, i.e., log(1+x)≈x. The exact expression for the CEU cooperation scheme for the resource constraint to perform better a MIMO downlink transmission scheme i.e., $C_{\mathrm{coop}}>C_{\mathrm{np}}$ is as follows:

(27) $$\begin{array}{ccc} C_{\mathrm{Cooperative}} & > & C_{\mathrm{Noncooperative}},\\ \frac{1}{2}\log_{2}\left(1+\lambda \;{\mathrm{SINR}}_{\mathrm{coop}}\right) & > & \log_{2}\left(1+\lambda \;{\mathrm{SINR}}_{\mathrm{nc}}\right),\\ \log_{2}\left(1+\lambda \;{\mathrm{SINR}}_{\mathrm{coop}}\right) & > & \log_{2}{\left(1+\lambda \;{\mathrm{SINR}}_{\mathrm{nc}}\right)}^{2},\\ \left(1+\lambda \;{\mathrm{SINR}}_{\mathrm{coop}}\right) & > & 1^{2}+{\left(\lambda \;{\mathrm{SINR}}_{\mathrm{nc}}\right)}^{2}+2\left(\lambda \;{\mathrm{SINR}}_{\mathrm{nc}}\right),\\ \left({\mathrm{SINR}}_{\mathrm{coop}}\right) & > & (\lambda \;{\mathrm{SINR}}_{\mathrm{nc}}^{2}+2\;{\mathrm{SINR}}_{\mathrm{nc}}). \end{array}$$

Therefore, it is sensible for the CEUs to decide whether to perform the cooperation downlink MIMO transmission scheme between adjacent CEUs.

### 4.3. Frequency Reuse (FR)

Frequency reuse (FR) is the reuse of the same frequency after a certain distance in cellular wireless systems. In general, a limited frequency bandwidth is divided into many subgroups on identifying the user location from the BS, where each group containing a few subcarriers is in turn assigned to adjacent cells. The fractional FR (FFR) and soft FR (SFR) techniques reduce the ICI coordination and aid in improving the spectral efficiency of CEU performance.

### 4.4. Fractional Frequency Reuse (FFR) and Soft Frequency Reuse (SFR)

In general, the FFR and SFR techniques divide a cell into inner and outer regions, and a different FR factor is used for each region. However, the distance from the BS to the CEUs can cause bad channel conditions and low quality of service (QoS); to solve these problems, locations of cell-centre (CC), cell-median (CM) and cell-edge (CE) users must be identified. In the downlink MIMO Gaussian interference channel, the **SINR** for *k* MUs in the *L* cell is given by

(28) $$\begin{array}{c} {\mathrm{SINR}}_{k,n}^{L}=\frac{P_{n}^{L}G_{k,n}^{L}}{\sum_{j\neq L}P_{n}^{L}G_{k,n}^{L}+\beta_{N}}, \end{array}$$

where $P_{n}^{L}$ is the downlink transmission power allocated by the BS, and $G_{k,n}^{L}$ is the channel gain for *K*th MUs and $\beta_{N}$ is the zero-mean additive white Gaussian noise (AWGN) power.

### 4.5. Identifying User Location

**Figure 5:**
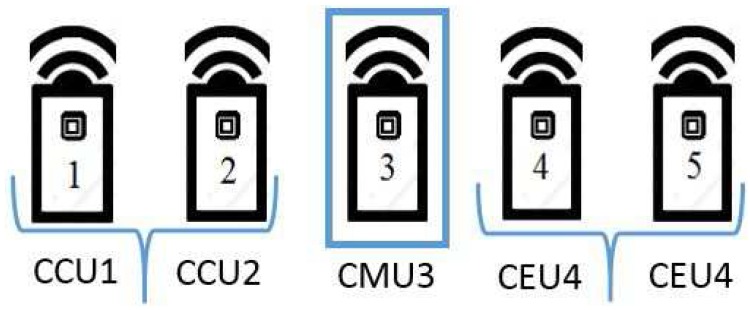
Identifying user location based on distance from base stations (BSs).

In the proposed scheme, base station (transmitter) is stationary and the mobile users (receiver) are in motion, and the efficient user pairing method always chooses the best users pair based on user location identification. In order to identify the user location based on distance from BSs, we split the users into three main groups, i.e., cell-center users (CCUs), cell-median users (CMUs), and cell-edge users (CEUs) with the help of median equation, in order to pair the users efficiently, as shown in Figure 5. If the receivers are in motion, users are separated based on the Euclidean norm of the channel vector and then the available users are split into three groups such as (CCUs, CMUs, CEUs) with the help of the median equation for the effective user pairing [22]. The efficient pairing constantly chooses the best pairing based on Euclidean norm and median equation; as a result, the proposed-partial cooperation CEUs scheme doesn’t affect the signal-to-interference plus noise ratio (SINR) and capacity drastically.

The major issue is to consider the interference from neighbouring cells because the CE users are significantly affected by ICI. Hence, the ICI signals must be identified and aligned to overcome the problem of low QoS and low throughput. The users near the BSs are considered as CCUs, the users with an average interference are considered as CMUs and the users with a higher interference from the neighbouring cells are considered as CEUs. We split the users into three main groups, i.e., CCUs, CMUs, and CEUs with the help of median, in order to pair the users efficiently. The CMUs are identified from the equation below:

(29) $$\begin{array}{c} \mathrm{CMUs}=\frac{∥{\hat{h}}_{\frac{K}{2}}∥+∥{\hat{h}}_{\frac{K}{2}+1}∥}{2}. \end{array}$$

By considering the CMU equation in Equation (29), CE users are identified. Coordination between the CEUs and ICI aid in improving the overall spectral efficiency of BS and MU performances. $MUs_{\left(4,5\right)}\Rightarrow \left\{u_{4},u_{5}\right\}$ from cell 1, $MUs_{\left(4,5\right)}\Rightarrow \left\{v_{4},v_{5}\right\}$ from cell 2 and $MUs_{\left(4,5\right)}\Rightarrow \left\{w_{4},w_{5}\right\}$ from cell 3. We consider the partial cooperation between CE MUs {$u_{5}$ and $v_{4}$} from cell 1 and cell 2, {$u_{4}$ and $w_{5}$} from cell 1 and cell 3 and {$v_{5}$ and $w_{4}$} from cell 2 and cell 3, respectively.

## 5. Partial-Cooperation between CE MUs and Power Analysis

In this section, we focus on the cooperative CE MUs and the power analysis. For L=3, the cooperation between the CEUs, capacity, power allocation and feasible conditions are discussed in the following three different cases.

### 5.1. Case 1: Partial-Cooperation between CE MUs {$u_{4}$ and $w_{5}$} from Cell 1 and Cell 3:

(30) $$\begin{array}{ccc} {\mathrm{SINR}}_{u_{4}} & = & \frac{P_{u_{4}}^{1}G_{u_{14}}}{\beta_{u_{4}}+P_{w_{5}}^{3}G_{w_{35}}}, \end{array}$$

(31) $$\begin{array}{ccc} {\mathrm{SINR}}_{W_{5}} & = & \frac{P_{w_{5}}^{3}G_{w_{35}}}{\beta_{w_{5}}+P_{u_{4}}^{1}G_{u_{14}}}. \end{array}$$

Consider ${\mathrm{SINR}}_{u_{4}}$ and ${\mathrm{SINR}}_{W_{5}}$ {$u_{4}$ and $w_{5}$} for the cooperation of users of cell 1 and cell 3, where $\beta_{u_{4}}=\frac{\sigma_{u_{4}}^{2}}{\sigma_{w_{5}}^{2}}$ and $\beta_{w_{5}}=\frac{\sigma_{w_{5}}^{2}}{\sigma_{u_{4}}^{2}}$. The total capacity of the downlink MIMO Gaussian interference channel for CEs partial cooperation users is given by

(32) $$\begin{array}{ccc} C_{\mathrm{coop}} & = & C_{u_{4}}+C_{w_{5}},\\ C_{\mathrm{coop}\{u_{4},w_{5}\}} & = & \frac{1}{2}\log_{2}\left(1+\frac{P_{w_{5}}^{3}G_{w_{35}}}{\beta_{w_{5}}+P_{u_{4}}^{1}G_{u_{14}}}\right)+\frac{1}{2}\log_{2}\left(1+\frac{P_{w_{5}}^{3}G_{w_{35}}}{\beta_{w_{5}}+P_{u_{5}}^{1}G_{u_{15}}}\right). \end{array}$$

The optimal power allocation for the partial cooperation of CEUs from cell 1 and cell 3 are $\left\{P_{u_{4}}^{1},P_{w_{5}}^{3}\right\}$ and the corresponding channel gains are $\{G_{u_{14}}$, $G_{w_{35}}\},$ respectively:

(33) $$\begin{array}{ccc} \left(P_{u_{4}}^{1},P_{w_{5}}^{3}\right) & = & \arg\max_{\{P_{u_{4}}^{1},P_{w_{5}}^{3}\}\in \Omega }\left\{C_{u_{4}}+C_{w_{5}}\right\}\\ & = & \arg\max_{\{P_{u_{4}}^{1},P_{w_{5}}^{3}\}\in \Omega }C_{\mathrm{coop}\{u_{4},w_{5}\}}, \end{array}$$

where $\Omega =\{P_{u_{4}}^{1},P_{w_{5}}^{3}|0\leq P_{u_{4}}^{1},P_{w_{5}}^{3}\leq \;P_{max}\}$ is the feasible set of $C_{\mathrm{coop}\{u_{4},w_{5}\}}$ CEUs.

### 5.2. Case 2: Partial-Cooperation between CE MUs {$u_{5}$ and $v_{4}$} from Cell 1 and Cell 2

(34) $$\begin{array}{ccc} {\mathrm{SINR}}_{u_{5}} & = & \frac{P_{u_{5}}^{1}G_{u_{15}}}{\beta_{u_{5}}+P_{v_{4}}^{2}G_{v_{24}}}, \end{array}$$

(35) $$\begin{array}{ccc} {\mathrm{SINR}}_{v_{4}} & = & \frac{P_{v_{4}}^{2}G_{v_{24}}}{\beta_{v_{4}}+P_{u_{5}}^{1}G_{u_{15}}}. \end{array}$$

Consider ${\mathrm{SINR}}_{u_{5}}$ and ${\mathrm{SINR}}_{v_{4}}$ {$u_{5}$ and $v_{4}$} for the partial cooperation of CE users in cell 1 and cell 2, where $\beta_{u_{5}}=\frac{\sigma_{u_{5}}^{2}}{\sigma_{v_{4}}^{2}}$ and $\beta_{v_{4}}=\frac{\sigma_{v_{4}}^{2}}{\sigma_{u_{5}}^{2}}$.

The total capacity achieved for partial cooperation of CE users is given below:

(36) $$\begin{array}{ccc} C_{\mathrm{coop}} & = & C_{u_{5}}+C_{v_{4}},\\ C_{\mathrm{coop}\{u_{5},v_{4}\}} & = & \frac{1}{2}\log_{2}\left(1+\frac{P_{u_{5}}^{1}G_{u_{15}}}{\beta_{u_{5}}+P_{v_{4}}^{2}G_{v_{24}}}\right)+\frac{1}{2}\log_{2}\left(1+\frac{P_{v_{4}}^{2}G_{v_{24}}}{\beta_{v_{4}}+P_{u_{5}}^{1}G_{u_{15}}}\right). \end{array}$$

The optimal power allocation for CEU partial cooperation in cell 1 and cell 2 is $\left\{P_{u_{5}}^{1},P_{v_{4}}^{2}\right\}$ and the corresponding channel gains are $\{G_{u_{15}}$, $G_{v_{24}}\}$, respectively:

(37) $$\begin{array}{ccc} \left(P_{u_{5}}^{1},P_{v_{4}}^{2}\right) & = & \arg\max_{\{P_{u_{5}}^{1},P_{v_{4}}^{3}\}\in \Omega }\left\{C_{u_{5}}+C_{v_{4}}\right\}\\ & = & \arg\max_{\{P_{u_{5}}^{1},P_{v_{4}}^{2}\}\in \Omega }C_{\mathrm{coop}\{u_{5},v_{4}\}}, \end{array}$$

where $\Omega =\{P_{u_{5}}^{1},P_{v_{4}}^{2}|0\leq P_{u_{5}}^{1},P_{v_{4}}^{2}\leq \;P_{max}\}$ is the feasible set of $C_{\mathrm{coop}\{u_{5},v_{4}\}}$ CEUs.

### 5.3. Case 3: Partial-Cooperation between CE MUs {$v_{5}$ and $w_{4}$} from Cell 2 and Cell 3

(38) $$\begin{array}{ccc} {\mathrm{SINR}}_{v_{5}} & = & \frac{P_{v_{5}}^{2}G_{v_{25}}}{\beta_{v_{5}}+P_{w_{4}}^{3}G_{w_{34}}}, \end{array}$$

(39) $$\begin{array}{ccc} {\mathrm{SINR}}_{v_{4}} & = & \frac{P_{w_{4}}^{3}G_{w_{34}}}{\beta_{w_{4}}+P_{v_{5}}^{2}G_{v_{25}}}. \end{array}$$

Consider ${\mathrm{SINR}}_{v_{5}}$ and ${\mathrm{SINR}}_{w_{4}}$ {$v_{5}$ and $w_{4}$} for partial cooperation of CE users in cell 2 and cell 3, where $\beta_{v_{5}}=\frac{\sigma_{v_{5}}^{2}}{\sigma_{w_{4}}^{2}}$ and $\beta_{w_{4}}=\frac{\sigma_{w_{4}}^{2}}{\sigma_{v_{5}}^{2}}$.

The total capacity achieved for partial cooperation of CE users is given below:

(40) $$\begin{array}{ccc} C_{\mathrm{coop}} & = & C_{v_{5}}+C_{w_{4}},\\ C_{\mathrm{coop}\{v_{5},w_{4}\}} & = & \frac{1}{2}\log_{2}\left(1+\frac{P_{v_{5}}^{2}G_{v_{25}}}{\beta_{v_{5}}+P_{w_{4}}^{3}G_{w_{34}}}\right)+\frac{1}{2}\log_{2}\left(1+\frac{P_{w_{4}}^{3}G_{w_{34}}}{\beta_{w_{4}}+P_{v_{5}}^{2}G_{v_{25}}}\right). \end{array}$$

The optimal power allocation for CEU partial cooperation in cell 2 and cell 3 is $\left\{P_{v_{5}}^{2},P_{w_{4}}^{3}\right\}$ and the corresponding channel gains are $\{G_{v_{25}}$, $G_{w_{34}}\}$, respectively:

(41) $$\begin{array}{ccc} \left(P_{v_{5}}^{2},P_{w_{4}}^{3}\right) & = & \arg\max_{\{P_{v_{5}}^{2},P_{w_{4}}^{3}\}\in \Omega }\left\{C_{v_{5}}+C_{w_{4}}\right\}\\ & = & \arg\max_{\{P_{v_{5}}^{2},P_{w_{4}}^{3}\}\in \Omega }C_{\mathrm{coop}\{v_{5},w_{4}\}}, \end{array}$$

where $\Omega =\{P_{v_{5}}^{2},P_{w_{4}}^{3}|0\leq P_{v_{5}}^{2},P_{w_{4}}^{3}\leq \;P_{max}\}$ is the feasible set of $C_{\mathrm{coop}\{v_{5},w_{4}\}}$ CEUs. To understand the general case and algorithms in Section 6, this redundant explanation for *K* = 5 users in Section 3 and *L* = 3 cells in Section 5 are very essential for the manuscript.

## 6. K-User, L-Multicell MIMO Gaussian Interference Channel through Staggered Antenna Switching and Power Analysis

**Figure 6:**
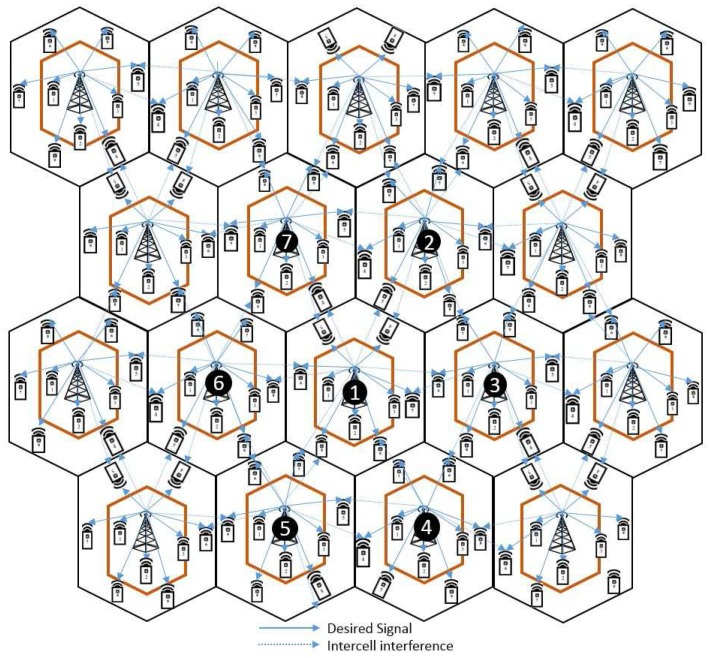
*K*-user MIMO Gaussian interference channel (K=k and L=l).

In this section, we explain the general case joint IA and power allocation for K∈{1,2,3,…,k} users, $s\in \{s_{1}^{\left[k\right]},s_{2}^{\left[k\right]},\ldots ,s_{n}^{\left[k\right]}\}$ beamforming vectors and L={1,2,…,ℓ} cells, described in Figure 6. Where we briefly explained the step by step procedure for the two main algorithms, *K*-user multiuser and multicell MIMO scheduling Algorithm 1 deliberates switching pattern and IA by cancelling similarly aligned dimensional subspace signals for K∈{1,2,3,…,k} users and $s\in \{s_{1}^{\left[k\right]},s_{2}^{\left[k\right]},\ldots ,s_{n}^{\left[k\right]}\}$ beamforming vectors, respectively. Algorithm 2 deliberates that *K*-user and *L*-cell edge user (CEUs) partial cooperation for L={1,2,…,ℓ} cells. We examined partial cooperation between CEUs into adjacent CEUs and power allocation for partial CEUs.
**Algorithm 1**
*K*-user Multiuser & Multicell MIMO Scheduling.Step:1.  Initialize: K∈{1,2,3,…,k}; $s\in \{s_{1}^{\left[k\right]},s_{2}^{\left[k\right]},\ldots ,s_{n}^{\left[k\right]}\}$;  ${\mathrm{SINR}}_{k,n}^{L}=\frac{P_{n}^{L}G_{k,n}^{L}}{\sum_{j\neq L}P_{n}^{L}G_{k,n}^{L}+\beta_{N}}$;$\tilde{P}=[\tilde{p_{1}},\tilde{p_{2}},\ldots ,\tilde{p_{K}}]=B^{(1/2)((K+1)+(K-1))\times K}.$Step:2.  Design:Switchingpattern&Channelmatrix:
1:**for** example: K={1,2,…,5}& $s=\{s_{1}^{\left[1\right]},s_{2}^{\left[1\right]}\}\;\mathrm{do}$
$$P^{T}=\left[\begin{array}{ccccc} 1 & 1 & 2 & 2 & 2\\ 2 & 1 & 2 & 2 & 1\\ 1 & 2 & 2 & 2 & 1\\ 2 & 2 & 2 & 1 & 1\\ 2 & 1 & 2 & 1 & 1 \end{array}\right]$$
i.e., $K=1;\;P_{1}^{T}=\left[\begin{array}{ccccc} 1 & 1 & 2 & 2 & 2 \end{array}\right];\;H_{1k}=\mathrm{diag}\left(\left[h_{1k}\left(1\right)\;h_{1k}\left(1\right)\;h_{1k}\left(2\right)\;h_{1k}\left(2\right)\;h_{1k}\left(2\right)\right]\right).$2:Beamforming vector: Two symbols per user $x_{1}^{\left[k\right]}=\left(s_{1}^{\left[k\right]},s_{2}^{\left[k\right]}\right)$.
$$s_{1}^{1}=\left[\begin{array}{ccccc} 1 & 1 & 0 & 0 & 1 \end{array}\right];\;s_{2}^{1}=\left[\begin{array}{ccccc} 0 & 1 & 1 & 1 & 0 \end{array}\right]$$3:Received signal vector for receiver 1: $y_{1}^{\left[1\right]}=H_{1k}\left(s_{1}^{\left[k\right]}+s_{1}^{\left[k\right]}\right)+Z_{1}$.
$$H_{11}\Rightarrow s_{1}^{1}=\left[\begin{array}{ccccc} h_{11}\left(1\right) & h_{11}\left(1\right) & 0 & 0 & h_{11}\left(2\right) \end{array}\right];\;H_{11}\Rightarrow s_{2}^{1}=\left[\begin{array}{ccccc} 0 & h_{11}\left(1\right) & h_{11}\left(2\right) & h_{11}\left(2\right) & 0 \end{array}\right];$$4:Aligning linear independent symbols: $H_{1k}\Rightarrow \left[h_{11},h_{12},h_{13},h_{14},h_{15}\right]$
$$R_{1}=\left[\begin{array}{ccccccc} h_{11}\left(1\right) & 0 & 0 & h_{13}\left(1\right) & h_{14}\left(2\right) & h_{14}\left(2\right) & h_{15}\left(2\right)\\ h_{11}\left(1\right) & h_{11}\left(1\right) & 0 & h_{13}\left(2\right) & h_{14}\left(2\right) & 0 & h_{15}\left(1\right)\\ 0 & h_{11}\left(2\right) & h_{12}\left(2\right) & h_{13}\left(2\right) & h_{14}\left(2\right) & 0 & 0\\ 0 & h_{11}\left(2\right) & h_{12}\left(2\right) & 0 & 0 & h_{14}\left(1\right) & 0\\ h_{11}\left(2\right) & 0 & h_{12}\left(1\right) & 0 & 0 & h_{14}\left(1\right) & h_{15}\left(1\right) \end{array}\right]$$5:Cancelling similar dimensional subspace signals:
$$\left[\begin{array}{c} \left(h_{11}=h_{12}\right),\left(h_{12}=h_{13}\right),\left(h_{14}=h_{15}\right) \end{array}\right];\;\left[\begin{array}{c} s_{2}^{\left[1\right]}=s_{1}^{\left[2\right]};\;s_{2}^{\left[2\right]}=s_{1}^{\left[3\right]};\;s_{2}^{\left[4\right]}=s_{1}^{\left[5\right]} \end{array}\right];$$
for fixed ${\mathrm{SINR}}_{k,n}^{L}$, $P^{T}$ and $\left\{s_{1}^{\left[k\right]},s_{2}^{\left[k\right]}\right\}$; compute K={1,2,…,5} and L={2,3} until aligned.   6:**end for**.Repeat the same approach for K∈{1,2,3,…,k} users ; $s\in \{s_{1}^{\left[k\right]},s_{2}^{\left[k\right]},\ldots ,s_{n}^{\left[k\right]}\}$ symbols and $\tilde{P}=[\tilde{p_{1}},\tilde{p_{2}},\ldots ,\tilde{p_{K}}]$ switching pattern for *K*-user.

We characterise the *K*-user multicell MIMO Gaussian interference channel through staggered antenna switching and power analysis for CE MUs in the absence of CSI. We restrict the total number of *K*-user communication pairs to be less than the number of transmitter M and receiver antennas N, where K≤M,N. We consider a fully connected *K*-user MIMO Gaussian interference channel, where the channel matrix and the transmit signal vector denoted by H, $x_{k}$ respectivelyand *K* independent users transmit *n* symbols is expressed as $s\in \{s_{1}^{\left[k\right]},s_{2}^{\left[k\right]},\ldots ,s_{n}^{\left[k\right]}\}$ . We design the antenna switching pattern by assuming a randomly generated transmission strategy for *K*-user $\tilde{P}=[\tilde{p_{1}},\tilde{p_{2}},\ldots ,\tilde{p_{K}}]$; however, we consider a low-cost reconfigurable multimode antenna switch among preset T=2 modes. Therefore, we can calculate the input and output relationship for desired and interference signals as follows.

(42) $$\begin{array}{cc} y_{k}\;\;\;\; & =\underset{\mathrm{Desired}\;\mathrm{signal}}{\underset{︸}{H_{11}x_{1}}}+\underset{\mathrm{Interference}\;+\;\mathrm{Noise}}{\underset{︸}{\;\sum_{K=2}^{k}H_{1K}x_{K}+Z_{k}}}=\left[\begin{array}{cc} h_{11}\left(1\right) & \vdots \\ h_{11}\left(2\right) & h_{11}\left(1\right)\\ \vdots & h_{11}\left(2\right)\\ \vdots & \vdots \\ h_{11}\left(2\right) & \vdots \end{array}\right]\;\;\left[\begin{array}{c} s_{1}^{\left[1\right]}\\ \vdots \\ \vdots \\ s_{n}^{\left[k\right]} \end{array}\right]+Z_{k}\\ & +\;\;\left[\begin{array}{cccccccc} \vdots & \vdots & \vdots & h_{13}\left(1\right) & h_{14}\left(2\right) & \cdots & h_{1k}\left(2\right) & h_{1k}\left(2\right)\\ h_{12}\left(1\right) & \vdots & \vdots & \vdots & h_{14}\left(2\right) & \cdots & 0 & \vdots \\ h_{12}\left(2\right) & h_{12}\left(2\right) & \vdots & h_{13}\left(2\right) & \vdots & \cdots & 0 & 0\\ \vdots & \vdots & h_{13}\left(2\right) & \vdots & \vdots & \cdots & h_{1k}\left(1\right) & 0\\ \vdots & h_{12}\left(1\right) & h_{13}\left(1\right) & 0 & 0 & \cdots & \vdots & h_{1k}\left(1\right) \end{array}\right]\left[\begin{array}{c} s_{1}^{\left[2\right]}\\ \vdots \\ s_{1}^{\left[3\right]}\\ \vdots \\ s_{1}^{\left[4\right]}\\ \vdots \\ s_{n}^{\left[k\right]} \end{array}\right] \end{array}$$

Algorithm 2 iterates over all *K*-users in a round-robin fashion. We can prove that the proposed algorithm converges for the partial cooperation of *K*-user and *L*-cell CEUs and allocates maximum power for all CEUs.

To produce the $s_{1}^{\left[k\right]}$ dimensional desired signal subspace, we must align $s_{n}^{\left[k\right]}$ dimensional interference signal subspaces and cancel the similar dimensional interference signals. Moreover, we propose the efficient Algorithm 1 for multiuser and multicell MIMO scheduling for the staggered antenna switching pattern. In Algorithm 2, we explain the CEU partial cooperation among *L* cells.
**Algorithm 2**
*K*-user and *L*-cell  Cell-Edge Users (CEUs) Partial Cooperation.Step:1.  Initialize:$L=\left\{1,2,\ldots ,\ell \right\};\;\;C_{\mathrm{nc}}=\log_{2}\left(1+\lambda \;{\mathrm{SINR}}_{\mathrm{nc}}\right);\;\;C_{\mathrm{coop}}=\alpha \log_{2}\left(1+\lambda \;{\mathrm{SINR}}_{\mathrm{coop}}\right);\;\;{\mathrm{SINR}}_{k,n}^{L}=\frac{P_{n}^{L}G_{k,n}^{L}}{\sum_{j\neq L}P_{n}^{L}G_{k,n}^{L}+\beta_{N}};\;\mathrm{CMUs}=\frac{∥{\hat{h}}_{\frac{K}{2}}∥+∥{\hat{h}}_{\frac{K}{2}+1}∥}{2};\;\;$Step:2.  Compare:CEUs(Cooperative&Noncooperative)Schemes:
1:**for** example: Cells L={1,2,3}do
$$\begin{array}{ccc} C_{\mathrm{Cooperative}} & > & C_{\mathrm{Noncooperative}}\\ \frac{1}{2}\log_{2}\left(1+\lambda \;{\mathrm{SINR}}_{\mathrm{coop}}\right) & > & \log_{2}\left(1+\lambda \;{\mathrm{SINR}}_{\mathrm{nc}}\right)\\ \left({\mathrm{SINR}}_{\mathrm{coop}}\right) & > & \left(\lambda \;{\mathrm{SINR}}_{\mathrm{nc}}^{2}+2\;{\mathrm{SINR}}_{\mathrm{nc}}\right) \end{array}$$2:Partial cooperation between CEUs into adjacent CEUs
$$i.e.,\;\;\left[u_{4}\;\&\;w_{5}\right];\;\;{\mathrm{SINR}}_{u_{4}}=\frac{P_{u_{4}}^{1}G_{u_{14}}}{\beta_{u_{4}}+P_{w_{5}}^{3}G_{w_{35}}};\;\;{\mathrm{SINR}}_{W_{5}}=\frac{P_{w_{5}}^{3}G_{w_{35}}}{\beta_{w_{5}}+P_{u_{4}}^{1}G_{u_{14}}}.$$
from [cell 1 & cell 3] as shown in Figure 43:   Total capacity of partial cooperative users:  $C_{\mathrm{coop}}=C_{u_{4}}+C_{w_{5}};$
$C_{\mathrm{coop}\{u_{4},w_{5}\}}=\frac{1}{2}\log_{2}\left(1+\frac{P_{w_{5}}^{3}G_{w_{35}}}{\beta_{w_{5}}+P_{u_{4}}^{1}G_{u_{14}}}\right)+\frac{1}{2}\log_{2}\left(1+\frac{P_{w_{5}}^{3}G_{w_{35}}}{\beta_{w_{5}}+P_{u_{5}}^{1}G_{u_{15}}}\right).$4:Optimal power allocation for partial CEUs
$$\left(P_{u_{4}}^{1},P_{w_{5}}^{3}\right)=\arg\max_{\{P_{u_{4}}^{1},P_{w_{5}}^{3}\}\in \Omega }C_{\mathrm{coop}\{u_{4},w_{5}\}}=\arg\max_{\{P_{u_{4}}^{1},P_{w_{5}}^{3}\}\in \Omega }\left\{C_{u_{4}}+C_{w_{5}}\right\}$$
where $\Omega =\{P_{u_{4}}^{1},P_{w_{5}}^{3}|0\leq P_{u_{4}}^{1},P_{w_{5}}^{3}\leq \;P_{max}\}$ is the feasible set $C_{\mathrm{coop}\{u_{4},w_{5}\}}$ CEUs.5:   for fixed ${\mathrm{SINR}}_{k,n}^{L}$; compute: $\left[u_{5},v_{4}\right]$ from [cell 1 & cell 2]; $\left[v_{5},w_{4}\right]$ from [cell 2 & cell 3] CEUs.   6:**end for**.Repeat the same approach for K∈{1,2,3,…,k} partial cooperative *K*-edge user.

## 7. Numerical Results

In this section, we present the simulation parameters in Table 4. The proposed five-user three-cell downlink MIMO Gaussian interference channel is shown in Figure 4, where we consider that MUs {1,2} are CCUs, MU 3 is a CMU and MUs {4,5} are CEUs, respectively.

**Figure 7:**
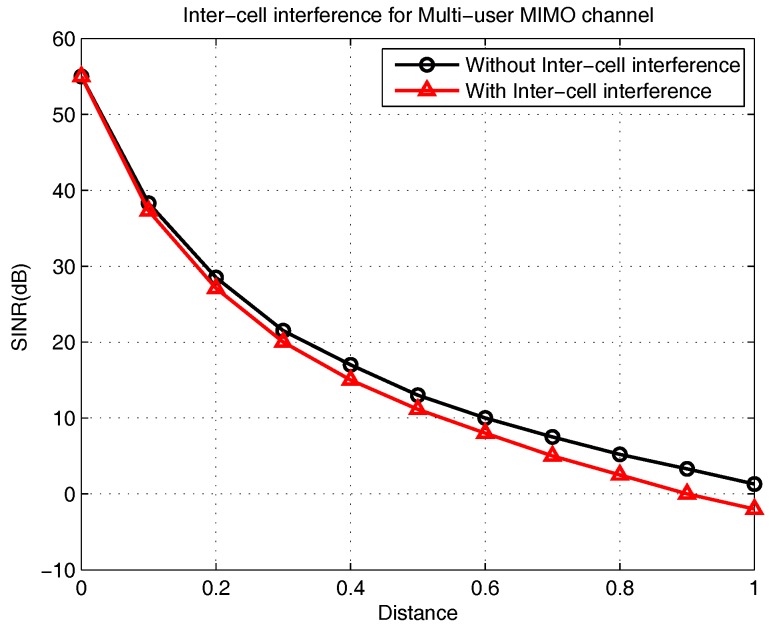
Intercell interference (ICI) for multiuser multiple-input-multiple-output channel.

In Figure 7, we evaluate the performance of the CEUs’ SINR (dB) versus the distance (km) with two main scenarios: with ICI and without ICI. Thus, the performance of the proposed scheme with ICI cooperation is compared to that without ICI cooperation. At an SINR of 10 dB and 0 dB without ICI, the SINR increases from 0.55 to 0.9 km, respectively, whereas in the proposed scheme with ICI, the SINR value varies from 0.6 to 1 km. The numerical results show that the ICI scheme achieves better performance compared to that without ICI. This drastic variation in SINR for the proposed scheme with ICI is expected, considering the cooperation between the CEUs from adjacent cells improve the CE MUs performance compared to the case without ICI. From the above discussion, we can accomplish that ICI has a larger impact on CEUs compared to that on CCUs and CMUs.

**Figure 8:**
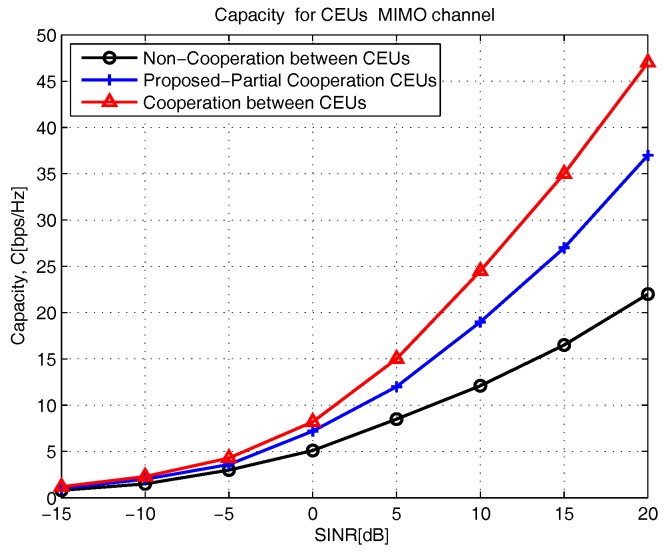
The capacity for cell-edge multiuser multiple-input-multiple-output interference channel.

Figure 8, presents the capacity C (bps/Hz) versus SINR (dB) with two main scenarios: cooperation between CEUs and noncooperation between CEUs. When the SINR increases from 5 (dB) to 20 (dB), the proposed partial cooperation CEUs scheme capacity increases from 12 C (bps/Hz) to 37.5 C (bps/Hz); for cooperation, the capacity increases from 9.5 C (bps/Hz) to 22 C (bps/Hz); however, for noncooperative CEUs, it varies from 15 C (bps/Hz) to 47.5 C (bps/Hz), respectively. This result is expected, and the overall MU performance improves by considering the full cooperation between neighbouring CE MUs. The proposed scheme operates partial-cooperation between CE MUs scheme and achieves better performance compared to non-cooperation CE MUs scheme and almost close performance compared to the full cooperation CE MUs scheme. This benefit comes from the partial cooperation of CE MUs such as $MUs_{\left(4,5\right)}\Rightarrow \left\{u_{4},u_{5}\right\}$ from cell 1, $MUs_{\left(4,5\right)}\Rightarrow \left\{v_{4},v_{5}\right\}$ from cell 2 and $MUs_{\left(4,5\right)}\Rightarrow \left\{w_{4},w_{5}\right\}$ from cell 3.

**Figure 9:**
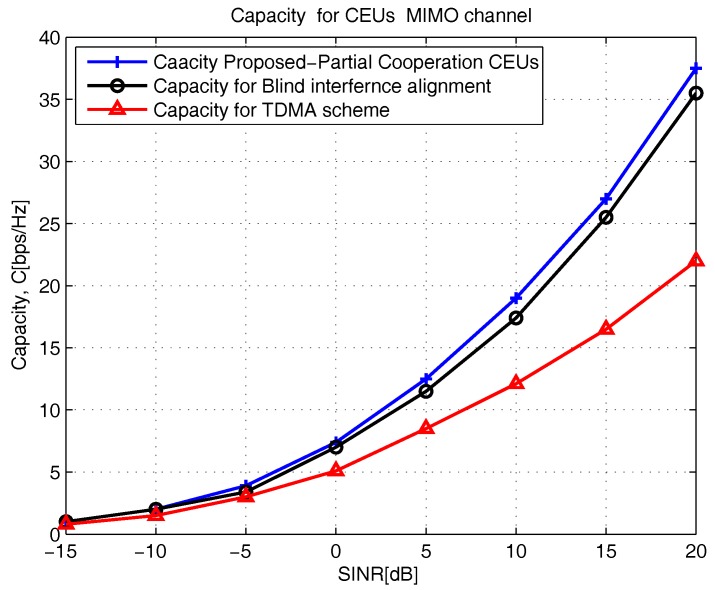
Comparison of Capacity with Partial, Blind and time division multiple access (TDMA) schemes.

Figure 9 shows the capacity performances of the proposed-partial cooperation between the CEU scheme is compared with blind-IA and time-division multiple access (TDMA) schemes [2]. As shown in Figure 9, when the SINR increases from 10 (dB) to 20 (dB), the capacity for the proposed-partial cooperation CEUs scheme increases from 19 C (bps/Hz) to 37.5 C (bps/Hz), whereas the blind interference alignment scheme capacity increases from 17.5 C (bps/Hz) to 35.5 C (bps/Hz) and TDMA scheme capacity increases from 12 C (bps/Hz) to 22 C (bps/Hz), respectively. This variation in capacity is predictable; the proposed-partial cooperation CEUs achieves better capacity than the blind-IA and time division multiple access (TDMA) schemes, since the proposed scheme constantly chooses the best user pairing based on Euclidean norm and median.

## 8. Conclusions

In this study, we aligned the interference signals using multimode staggered antenna mode switching on the receiver. A key insight behind the proposed alignment scheme is that any vector aligned at one undesired receiver cannot be aligned at another unintended receiver because the similar dimensional subspace signals are cancelled to align the desired and interference signals in an efficient manner. Moreover, we computed the power allocation and feasibility condition for cooperative CE MUs in the absence of CSI knowledge. The new approach (randomly generated transmission strategy) effectively aligned and eliminated the similar dimensional subspace signals. Importantly, the cooperation between CEUs for a multicell MIMO Gaussian interference channel eliminates ICI and improves the overall system performance drastically. In addition, the numerical results showed that the proposed intercell interference scheme with partially-cooperative CE MUs enhances the capacity and SINR performance compared to noncooperative CE MUs and without intercell interference schemes, respectively.

## Figures and Tables

**Table 1:** Antenna switching pattern for receiver 1.

At Receiver 1					
$H_{11}\;⟶\;s_{1}^{1}$	$h_{11}\left(1\right)$	$h_{11}\left(1\right)$	0	0	$h_{11}\left(2\right)$
$H_{11}\;⟶\;s_{2}^{1}$	0	$h_{11}\left(1\right)$	$h_{11}\left(2\right)$	$h_{11}\left(2\right)$	0
$H_{12}\;⟶\;s_{1}^{2}$	0	$h_{12}\left(1\right)$	$h_{12}\left(2\right)$	$h_{12}\left(2\right)$	0
$H_{12}\;⟶\;s_{2}^{2}$	0	0	$h_{12}\left(2\right)$	$h_{12}\left(2\right)$	$h_{12}\left(1\right)$
$H_{13}\;⟶\;s_{1}^{3}$	0	0	$h_{13}\left(2\right)$	$h_{13}\left(2\right)$	$h_{13}\left(1\right)$
$H_{13}\;⟶\;s_{2}^{3}$	$h_{13}\left(1\right)$	$h_{13}\left(2\right)$	$h_{13}\left(2\right)$	0	0
$H_{14}\;⟶\;s_{1}^{4}$	$h_{14}\left(2\right)$	$h_{14}\left(2\right)$	$h_{14}\left(2\right)$	0	0
$H_{14}\;⟶\;s_{2}^{4}$	$h_{14}\left(2\right)$	0	0	$h_{14}\left(1\right)$	$h_{14}\left(1\right)$
$H_{15}\;⟶\;s_{1}^{5}$	$h_{15}\left(2\right)$	0	0	$h_{15}\left(1\right)$	$h_{15}\left(1\right)$
$H_{15}\;⟶\;s_{2}^{5}$	$h_{15}\left(2\right)$	$h_{15}\left(1\right)$	0	0	$h_{15}\left(1\right)$

**Table 2:** Antenna switching pattern for receiver 2.

At Receiver 3					
$H_{33}\;⟶\;s_{1}^{3}$	0	0	$h_{33}\left(2\right)$	$h_{33}\left(2\right)$	$h_{33}\left(1\right)$
$H_{33}\;⟶\;s_{2}^{3}$	$h_{33}\left(1\right)$	$h_{33}\left(2\right)$	$h_{33}\left(2\right)$	0	0
$H_{31}\;⟶\;s_{1}^{1}$	$h_{31}\left(1\right)$	$h_{31}\left(1\right)$	0	0	$h_{31}\left(2\right)$
$H_{31}\;⟶\;s_{2}^{1}$	0	$h_{31}\left(1\right)$	$h_{31}\left(2\right)$	$h_{31}\left(2\right)$	0
$H_{32}\;⟶\;s_{1}^{2}$	0	$h_{32}\left(1\right)$	$h_{32}\left(2\right)$	$h_{32}\left(2\right)$	0
$H_{32}\;⟶\;s_{2}^{2}$	0	0	$h_{32}\left(2\right)$	$h_{32}\left(2\right)$	$h_{32}\left(1\right)$
$H_{34}\;⟶\;s_{1}^{4}$	$h_{34}\left(2\right)$	$h_{34}\left(2\right)$	$h_{34}\left(2\right)$	0	0
$H_{34}\;⟶\;s_{2}^{4}$	$h_{34}\left(2\right)$	0	0	$h_{34}\left(1\right)$	$h_{34}\left(1\right)$
$H_{35}\;⟶\;s_{1}^{5}$	$h_{35}\left(2\right)$	0	0	$h_{35}\left(1\right)$	$h_{35}\left(1\right)$
$H_{35}\;⟶\;s_{2}^{5}$	$h_{35}\left(2\right)$	$h_{35}\left(1\right)$	0	0	$h_{35}\left(1\right)$

**Table 3:** Antenna switching pattern for receiver 3.

At Receiver 5					
$H_{55}\;⟶\;s_{1}^{5}$	$h_{55}\left(2\right)$	0	0	$h_{55}\left(1\right)$	$h_{55}\left(1\right)$
$H_{55}\;⟶\;s_{2}^{5}$	$h_{55}\left(2\right)$	$h_{55}\left(1\right)$	0	0	$h_{55}\left(1\right)$
$H_{51}\;⟶\;s_{1}^{1}$	$h_{51}\left(1\right)$	$h_{51}\left(1\right)$	0	0	$h_{51}\left(2\right)$
$H_{51}\;⟶\;s_{2}^{1}$	0	$h_{51}\left(1\right)$	$h_{51}\left(2\right)$	$h_{51}\left(2\right)$	0
$H_{52}\;⟶\;s_{1}^{2}$	0	$h_{52}\left(1\right)$	$h_{52}\left(2\right)$	$h_{52}\left(2\right)$	0
$H_{52}\;⟶\;s_{2}^{2}$	0	0	$h_{52}\left(2\right)$	$h_{52}\left(2\right)$	$h_{52}\left(1\right)$
$H_{53}\;⟶\;s_{1}^{3}$	0	0	$h_{53}\left(2\right)$	$h_{53}\left(2\right)$	$h_{53}\left(1\right)$
$H_{53}\;⟶\;s_{2}^{3}$	$h_{53}\left(1\right)$	$h_{53}\left(2\right)$	$h_{53}\left(2\right)$	0	0
$H_{54}\;⟶\;s_{1}^{4}$	$h_{54}\left(2\right)$	$h_{54}\left(2\right)$	$h_{54}\left(2\right)$	0	0
$H_{54}\;⟶\;s_{2}^{4}$	$h_{54}\left(2\right)$	0	0	$h_{54}\left(1\right)$	$h_{54}\left(1\right)$

**Table 4:** Simulation parameters.

Parameters	Values
Number of cells and users	L=3 and K=5
Cell shape	Hexagonal cell
User location	Cell-center users(CCUs),Cell-median users(CMUs),Cell-edge users(CEUs)
Identified user positions	CCUs = MUs{1,2}, CMUs= MU {3}, CEUs = MUs{4,5}
Number of base station antennas and users	7, 3
Bandwidth and carrier Frequency	10 MHz and 2 GHz
Channel fading and log-normal shadowing	i.i.d Gaussian distribution and Rayleigh fading

## Abbreviations

The following abbreviations are used in this manuscript:

FR	frequency reuse
MUs	mobile users
IA	interference alignment
CE	cell-edge
CC	cell-center
CM	cell-median
BSs	base stations
DoF	degree of freedom
AWGN	additive white Gaussian noise power
MIMO	multiple-input-multiple-output
MISO	multiple-input-single-output
SISO	single-input-single-output
R-STIA	relay space-time interference alignment
STIA	space-time interference alignment
CSIR	channel state information at the relay
CSIT	channel state information at the transmitter
CSI	channel state information
ICI	intercell interference
IUI	interuser interference
CEUs	cell-edge users
CCUs	cell-center users
CMUs	cell-median users
BER	bit-error rate
FFR	fractional frequency reuse
SFR	soft frequency reuse
BER	bit-error rate
TDMA	time-division multiple access
SINR	signal-to-interference plus noise ratio

## References

[1] Karmakar S., Varanasi M.K. (2013). The capacity region of the MIMO interference channel and its reciprocity to within a constant gap. IEEE Trans. Inf. Theory.

[2] Xiao C., Zaichen Z., Lili Z., Liang W., Du J., Lu P.-S., Sun C. (2017). Blind Interference Alignment in Two-Cell Z Interference MIMO Channel. IEEE Access.

[3] Shin W., Lee N., Lee J., Poor H.V. Guiding blind transmitters for *K*-user MISO interference relay channels with Imperfect channel knowledge. Proceedings of the International Symposium on Information Theory (ISIT).

[4] Selvaprabhu P., Chinnadurai S., Li J., Lee M.H. (2017). Topological Interference Management for *K*-user Downlink Massive MIMO Relay Network Channel. Sensors.

[5] Jin J., Gao X.C., Li X., Li S., Wang Z. (2017). Achievable Degrees of Freedom for the Two-Cell Two-Hop MIMO Interference Channel With Half-Duplex Relays. IEEE Access.

[6] Maleki H., Jafar A.S., Shamai S. (2012). Retrospective interference alignment over interference networks. IEEE J. Sel. Top. Signal Process..

[7] Sun H., Jafar S.A. Blind interference alignment for private information retrieval. Proceedings of the IEEE International Symposium on Information Theory (ISIT).

[8] Hai H., Jiang X., Lee M.H., Jeong Y. (2017). Efficient Transmission and Detection Based on RNS for Generalized Space Shift Keying. IEEE Wirel. Commun. Lett..

[9] Sung H., Park S.-H., Lee K.-J., Lee I. (2010). Linear precoder designs for *K*-user interference channels. IEEE Trans. Wirel. Commun..

[10] Kang M.G., Choi W. (2013). Ergodic interference alignment with delayed feedback. IEEE Signal Process. Lett..

[11] Gou T., Wang C., Jafar S.A. (2011). Aiming perfectly in the dark-blind interference alignment through staggered antenna switching. IEEE Trans. Signal Process..

[12] Alaa A.M., Ismail M.H. (2017). Achievable Degrees of Freedom of the *K*-user SISO Interference Channel With Blind Interference Alignment Using Staggered Antenna Switching. IEEE Trans. Veh. Technol..

[13] Lee K.J., Sung H., Park E., Lee I. (2010). Joint optimization for one and two-way MIMO AF multiple-relay systems. IEEE Trans. Wirel. Commun..

[14] Jafar S.A. Exploiting channel correlations-simple interference alignment schemes with no CSIT. Proceedings of the IEEE Global Telecommunications Conference (GLOBECOM 2010).

[15] Abuibaid M.A., Çolak S.A. (2017). Energy-efficient massive MIMO system: Exploiting user location distribution variation. AEU Int. J. Electron. Commun..

[16] Wang C., Gou T., Jafar S.A. Interference alignment through staggered antenna switching for MIMO BC with no CSIT. Proceedings of the 2010 Conference Record of the Forty Fourth Asilomar Conference on Signals, Systems and Computers.

[17] Sudarshan P., Mehta N.B., Molisch A.F., Zhang J. Antenna selection with RF pre-processing: Robustness to RF and selection non-idealities. Proceedings of the IEEE Radio Wireless Conference.

[18] Li Z., Xia X.G. (2007). A simple Alamouti space time transmission scheme for asynchronous cooperative systems. IEEE Signal Process. Lett..

[19] Wang C. (2014). Degrees of freedom characterization: The 3-user SISO interference channel with blind interference alignment. IEEE Commun. Lett..

[20] Ying T., Feng W., Su W., Jiang W. (2017). On the Degrees of Freedom of MIMO X Networks With Non-Cooperation Transmitters. IEEE Trans. Wirel. Commun..

[21] Etkin R., Ordentlich E. On the degrees-of-freedom of the *K*-user Gaussian interference channel. Proceedings of the IEEE International Symposium on Information Theory.

[22] Chinnadurai S., Selvaprabhu P., Jeong Y., Sarker A.L., Hai H., Duan W., Lee M.H. (2017). User Clustering and Robust Beamforming Design in Multicell MIMO-NOMA System for 5G Communications. AEU Int. J. Electron. Commun..

